# Identification and Spatial Analysis of Land Salinity in China’s Yellow River Delta Using a Land Salinity Monitoring Index from Harmonized UAV-Landsat Imagery

**DOI:** 10.3390/s23177584

**Published:** 2023-09-01

**Authors:** Liping Jiang, Guanghui Qiu, Xinyang Yu

**Affiliations:** 1 Shandong Geological Exploration Institute of China Chemical Geology and Mine Bureau, Jinan 250013, China; 2 College of Resources and Environment, Shandong Agricultural University, Tai’an 271018, China; 3 Institute of Geographic Sciences and Natural Resources Research, Chinese Academy of Sciences, Beijing 100101, China

**Keywords:** land salinity retrieval, remote sensing, spatial analysis, random forest, Landsat-9 OLI

## Abstract

Precise identification and spatial analysis of land salinity in China’s Yellow River Delta are essential for the rational utilization and sustainable development of land resources. However, the accurate retrieval model construction for monitoring land salinity remains challenging. This study constructed a land salinity retrieval framework using a harmonized UAV and Landsat-9 multi-spectral dataset. The Kenli district of the Yellow River Delta was selected as the case study area, and a land salinity monitoring index (LSMI) was proposed based on field survey data and UAV multi-spectral image and applied to the reflectance-corrected Landsat-9 OLI image. The land salinity distribution patterns were then mapped and spatially analyzed using Moran’s I and Getis-Ord GI* analysis. The results demonstrated the following: (1) The LSMI-based method can accurately retrieve land salinity content with a validation determination coefficient (*R*^2^), root mean square error (*RMSE*), and residual predictive deviation (*RPD*) of 0.75, 1.89, and 2.11, respectively. (2) Land salinization affected 93.12% of the cultivated land in the study area, and the severely saline soil grade (with a salinity content of 6–8 g/kg) covered 38.41% of the total cultivated land area and was widely distributed throughout the study area. (3) Saline land exhibited a positive spatial autocorrelation with a value of 0.311 at the *p* = 0.000 level; high–high cluster types occurred mainly in the Kendong and Huanghekou towns (80%), while low–low cluster types were mainly located in the Dongji, Haojia, Kenli, and Shengtuo towns (88.46%). The spatial characteristics of various salinity grades exhibit significant variations, and conducting separate spatial analyses is recommended for future studies.

## 1. Introduction

Land salinization has significant implications for the ecological environment at a worldwide scale [1,2]. Over 1 billion hectares or approximately 10% of the world’s total land resources are at risk of salinization [3,4]. In China, the area of land resources affected by salinization is more than 36.3 million hectares [5], mainly distributed in arid and semi-arid areas [6] and coastal areas (e.g., the Yellow River Delta) [7,8]. By incorporating the management and planning of saline land resources into the national food security strategic system, China has designated such lands as future reserve cultivated land resources [9]. Precise identification and mastery of the spatial distribution characteristics of saline land, as well as a quantitative description and spatial analysis of land salinization levels, are crucial for optimizing land resource allocation, maintaining ecosystem health, and promoting regional sustainable development.

The causes of land salinization can be categorized into two types: natural salinization and anthropogenic salinization [10]. Capillary action or evapotranspiration may lead to the rise of groundwater and the accumulation of soluble salts on the soil surface, resulting in varying degrees of salinity [11]. Land salinization typically occurs in regions characterized by arid climates, high rates of evapotranspiration, shallow water tables, and elevated levels of soluble salts. This phenomenon can lead to a significant reduction in soil productivity and biodiversity, as well as an imbalance in the soil’s acid–base equilibrium and deterioration of regional ecosystems [12,13], which has become a major environmental issue that hinders social and economic development and threatens the ecological environment. As a complex and dynamic system, soil changes over time and space [14,15]. Therefore, it is crucial to develop effective methods for monitoring the extent of regional land salinization and uncovering its distribution patterns.

The conventional approaches to land salinity measurement contain field surveys and electrical conductivity measurements, which are theoretically accurate but require significant time and labor resources [16,17]. Moreover, this method does not allow for the monitoring of spatial distribution patterns in land salinity content. The introduction of satellite remote-sensing technology enables a broad detection range and high acquisition efficiency, thereby facilitating the provision of spectral information on land salinization at short intervals [18]. By establishing predictive models that correlate remotely sensed soil salt data with ground monitoring, relatively small sample-size verification data are required for assessing land salinization on the ground, which helps reduce monitoring costs. Scholars therefore have utilized RS images and corresponding indexes to investigate and monitor land salinity. For instance, Azabdaftari et al. (2016) computed vegetation indexes to retrieve land salinity in Turkey using Landsat multi-spectral images from four different intervals [19]. Morgan et al. (2018) forecasted land salinity in Cairo, Egypt, using Sentinel-2 multi-spectral data and neural network classification methods [20]. Wang et al. (2021) combined Sentinel-2 and three machine-learning methods to estimate and map the land salinity in arid areas of China [21]. Ge et al. (2022) used Sentinel-2 image, environmental covariates, and hybrid machine-learning approaches to update land salinity with fine spatial resolution and high accuracy [22]. Kaplan et al. (2023) predicted land salinity using machine learning and Sentinel-2 data in hyper-arid areas [23]. Alamda et al. (2023) detected land salinity using Lansat-8 OLI image and machine-learning algorithms [24]. All the studies found that it could be possible to estimate soil salinity to an excellent extent by satellite data. However, the accurate monitoring of soil salinization is constrained by the spatial resolution limitations of satellite remote-sensing images (10–50 m), necessitating the urgent acquisition of high-resolution imagery to provide enhanced support.

Different from satellite RS means, unmanned aerial vehicle (UAV) spectral sensors are highly maneuverable and have been used as an essential data source to monitor land salinity since the 2010s. Ivushkin et al. (2019) investigated the plot-scale assessment of land salinity using three different UAV-mounted sensors [25]. Zhao et al. (2021) developed and optimized an inversion monitoring model for monitoring soil salt content using UAV multi-spectral remote-sensing data and a backpropagation neural network in northwest Oasis China [26]. Yang et al. (2021) examined the effect of spring irrigation on land salinity monitoring with a UAV multi-spectral sensor, and found that accurate regional salinity maps could be plotted based on the spectral indices selected by a genetic algorithm [27]. Yu et al. (2022) proposed a soil salinity retrieval index to investigate the feasibility of the UAV sensor of Sequoria to inverse soil salinity [28]. Studies have indicated that the index in the visible-to-infrared spectrum may better measure land salinity, which can increase the accuracy of land salinity retrieval. However, UAVs alone cannot detect and monitor land salinity at a regional scale. To boost the spectral resolution to retrieve land salinity, Xie et al. combined Sentinel-2A and UAV multi-spectral images to increase the spectral resolution to retrieve regional land salinity [29]. Qi et al. (2021) retrieved land salinity in coastal corn planting areas using the Sentinel-2A satellite–UAV–ground integration approach, and found that the use of satellite and UAV images can improve the retrieval accuracy of land salinity [30]. Even though scholars have tested the ability of land salinity monitoring using Sentinel-2A satellite and UAV images, an in-depth study is essential for the construction of a reliable land salinity retrieval index based on Landsat-9 OLI and UAV images due to the longer time coverage and stability provided by Landsat imagery.

This study selected Kenli District in the Yellow River Delta as the case study area. The aims are to (1) construct monitoring models of the land salinity content based on UAV imagery and field-measured data, (2) construct the relationship between the reflectance of UAV and Landsat-9 OLI satellite images to normalize the reflectance of satellite image, (3) apply the optimal monitoring model to the normalized satellite imagery to achieve scaled-up land salinity monitoring method, and (4) explore the spatial distribution patterns of various grades of salinity soil at a regional scale.

## 2. Study Area

The study was conducted in the representative cultivated land region of the Kenli district, YRD (37°35′6″~37°35′14″ N, 118°20′31″~118°20′46″ E). The study area contains 9 towns, i.e., Dongying Demonstration Zone (DDZ hereinafter), Dongji Town (DJ), Haojia (HJ), Huanghekou (HHK), Kendong (KD), Kenli (KL), Shengtuo (ST), Xinglong (XL), and Yong’an (YA) with a total area of 1246.51 km^2^, in which cultivated land covers 894.34 km^2^. The terrain in the study area is gently sloping with typical alluvial plain landforms. The study area features a temperate continental monsoon climate that is characterized by dry and windy conditions during spring. The potential evapotranspiration–precipitation ratio in the study area is higher than 7, resulting in limited vegetation coverage and severe salt deposition in the soil. The main soil types in the study area are coastal saline alkaline soil and fluvo-aquic soils. The groundwater table has a shallow depth and high mineral content. The cultivated lands in the study area cover 894.34 km^2^, which is the predominant land-use type.

## 3. Methodology

### 3.1. Data Processing

In the study area, the spring season is characterized by high evapotranspiration rates and land salinity accumulation, which is critical for the growth of crops planted on the cultivated land. Therefore, referring to the revisit time of the Landsat-9 satellite and the local weather conditions, a field survey was conducted on 16 April 2023 to collect soil samples and fly UAV to obtain UAV multi-spectral images.

**Figure 1:**
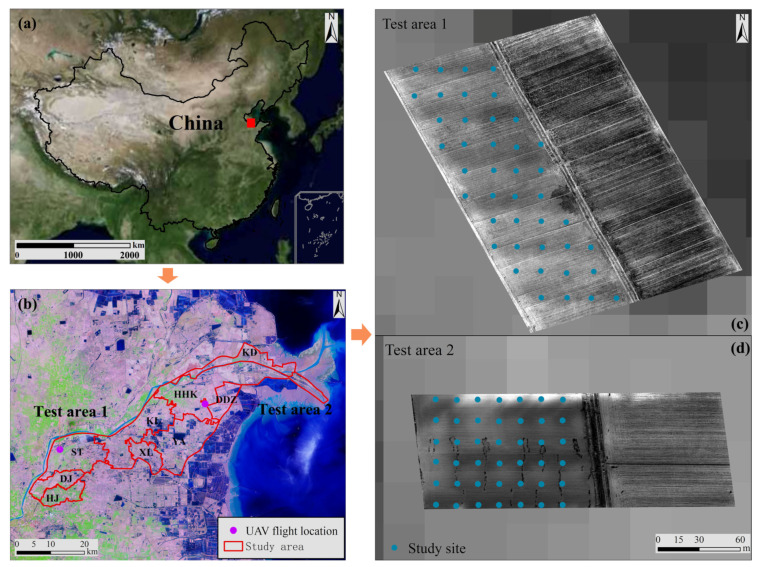
Location of the study area: (**a**) Kenli district in China; (**b**) test areas in the Kenli district (the base image is the false-color Landsat-9 OLI image); (**c**,**d**) UAV image covering the test area.

Two experimental plots were established on the cultivated land in the study area to fly the UAV system (DJI M600PRO + Sequoria multi-spectral sensor). Test Area 1 encompassed an area of 3.78 ha and was planted with winter wheat. As April is the jointing period of winter wheat, the vegetation coverage in the test area was relatively low. Test Area 2, which covered an area of 1.89 ha, was left fallow after harvest (see Figure 1). Eighty ground control points were evenly distributed throughout the test areas, and measurements were taken using an EC110 portable salinity meter equipped with a 2225FST series probe (with temperature correction for electrical conductivity) from Spectrum Technologies Inc. (Dallas/Fort Worth, TX, USA). More details about the field work, UAV flight set, and soil sample processing can be found in [28].

The Landsat-9 OLI image covering the study area was acquired on 16 April 2023 from the United States Geological Survey (http://earthexplorer.usgs.gov/, accessed on 26 April 2023). Radiometric calibration, fast line-of-sight atmospheric analysis of spectral hypercubes (FLAASH) atmospheric correction, geometry correction, and Gram–Schmidt Pan Sharpening were conducted to obtain a 15 m resolution surface reflectance image using an IDL program [31]. Table 1 presents the spectral band information of multi-spectral sensors, including UAV and Landsat-9 OLI, within the wavelength range of 550 to 865 nm.

### 3.2. Model Construction and Validation

As found in our previous study, G, R, and NIR are significantly sensitive to land salinity [28]. In this study, the reflectance of the sensitive band underwent mathematical transformations or combinations through algebraic computations such as addition, subtraction, division, and logarithmic or reciprocal transformation to construct land salinity retrieval models. Additionally, ratios were taken by combining addition and division or their reciprocals (Table 2). Individual or combined bands with |R| > 0.45 were further selected as sensitive parameters for screening purposes.

To evaluate the performance of the newly proposed index, commonly used land salinity retrieval indexes, including a vegetation index and a salinity index, were utilized for validation processes. The vegetation index was derived from standard multi-spectral remote-sensing bands R and NIR, encompassing the normalized difference vegetation index (NDVI, Equation (1)). The salinity index of the soil remote-sensing index (SRSI) refers to the land salinity level and is represented by Equation (2).

(1) $$\mathrm{NDVI}=\frac{NIR-R}{NIR+R}$$

(2) $$\mathrm{SRS}I=\sqrt{{\left(\mathrm{NDVI}-1\right)}^{2}+{\mathrm{SI}1}^{2}}\;\mathrm{SI}1=\sqrt{\begin{array}{c} G\times R \end{array}}$$

To evaluate the performance of the proposed index, the determination coefficient (*R*^2^), root mean square error (*RMSE*), and residual predictive deviation (*RPD*) were utilized to assess the regression outcomes. *R*^2^ indicates the consistency of model establishment and validation. A high value of *R*^2^ (e.g., 1) denotes that the model is more robust and has a better fitting degree. The *RMSE* serves as a metric for assessing the predictive performance of a model, with lower values indicating superior prediction capabilities. The *RPD* represents the ratio between the standard deviation of measured values and predicted errors. Models with high *R*^2^ and *RPD* values exhibit superior performance in terms of both prediction accuracy and stability [32].

### 3.3. Image Correction

To apply Landsat-9 data to the land salinity monitoring model and investigate regional-scale land salinization, the UAV multi-spectral data were utilized to correct the reflectance of the Landsat-9 multi-spectral image. To ensure the feasibility of correcting Landsat-9 images based on UAV images, the average reflectance of the UAV-sensitive band of all sampling points and the corresponding sensitive bands (green, red, and near-infrared) of Landsat-9 image were calculated, and then the average reflectance variation trend of the three bands was compared to depict the scatter plot of the average reflectance of the corresponding bands. Subsequently, the ratio correction method was employed to normalize the reflectivity of Landsat-9 images [33]. For instance, the ratio between the near-infrared reflectance of the Landsat-9 image and the near-infrared reflectance of the fitted UAV image was calculated, and then the average of all ratios was calculated as the near-infrared reflectance correction coefficient. The reflectance correction coefficients of other bands were computed by the same method. Finally, the reflectances of sensitive bands of the Landsat-9 image were corrected, which constructed the harmonized UAV-Landsat image dataset.

### 3.4. Spatial Analysis

The spatial patterns of land salinity in the study area were analyzed using Moran’s I and Getis-Ord GI* analysis. The global Moran index is a metric that quantifies the overall spatial clustering of data [34]. If the global Moreland index is significant, it can be considered that there is a spatial correlation in this region. The global Moran’s I is defined as:

(3) $$I=\frac{N}{W}\frac{\sum_{i=1}^{N}\sum_{j=1}^{N}w_{ij}(x_{i}-\bar{x})(x_{j}-\bar{x})}{\sum_{j=1}^{N}{\left(x_{i}-\bar{x}\right)}^{2}}$$

where I is the global Moran‘s I, N represents the number of spatial units indexed by i and j, x is the variable of interest, $\bar{x}\;$ is the mean of x, $w_{ij}\;$ is the spatial weight between feature i and j, and W is the sum of all $w_{ij}$.

However, it is still unknown where the phenomenon of spatial aggregation exists in specific places. The local Moran index measures the degree of spatial correlation between each spatial object and its neighboring objects within the analysis region [35]. The computation equation is shown below.

(4) $$I_{i}=\frac{Z_{i}}{S^{2}}\sum_{j\neq i}^{n}w_{ij}Z_{j}$$

where $I_{i}$ is the local Moran index, $Z_{i}$*=*$x_{i}-\bar{x}$, $Z_{j}$=$x_{j}-\bar{x}$, $S^{2}$*=*$\frac{\sum {\left(x_{i}-\bar{x}\right)}^{2}}{n}$, n represents the number of spatial units, x is the variable of interest, $\bar{x}$ is the mean of x, $w_{ij}$ is the spatial weight between feature i and j.

The hot-spot analysis tool computes the Getis-Ord Gi* statistic for each feature in a dataset, providing an effective means to investigate local spatial clustering distribution characteristics that can differentiate variable spatial distributions into cold and hot spots [36]. The Getis-Ord Gi* statistic is computed using the following equations.

(5) $$G_{i}^{*}=\frac{\sum_{j=1}^{n}w_{i,j}x_{j}-\bar{X}\sum_{j=1}^{n}w_{i,j}}{S\sqrt{\frac{\left[n\sum_{j=1}^{n}w_{i,j}^{2}-{\left(\sum_{j=1}^{n}w_{i,j}\right)}^{2}\right]}{n-1}}}\bar{X}=\frac{\sum_{j=1}^{n}w_{i,j}}{n}S=\sqrt{\frac{\sum_{j=1}^{n}x_{j}^{2}}{n}-({\bar{X})}^{2}}$$

where n is the total number of features, $x_{j}$ is the attribute value for feature j, and $w_{i,j}$ is the spatial weight between feature i and j.

## 4. Results

### 4.1. Retrieval Model Construction

Various combinations of the three land-salinity-sensitive bands (R, G, and NIR) were compared and the sensitive parameters (|R| > 0.45) were filtered in Table 3. For the single sensitive band information, NIR showed the highest correlation with land salinity content. In the division section, NIR/R exhibited a |R| of 0.58 with salinity content. In the ratio section, |(R − NIR)/(NIR + G)| had a |R| of 0.63. In order to simplify the equation and make it more applicable, the absolute value symbols were removed, and the order of R and NIR was adjusted to be consistent with the order of the denominators. Therefore, a new index, namely the land salinity monitoring index (LSMI, Equation (4)), can be devised to detect land salinity by relying on the three sensitive bands.

(6) $$\mathrm{LSM}I=\frac{NIR-R}{NIR+G}$$

where G, R, and NIR are the green, red, and near-infrared band reflectance of the UAV image, respectively.

### 4.2. Validation

The correlations of proposed LSMI, NDVI, and SRSI with land salinity content are shown in Table 4. The comparison found that LSMI showed the highest Gray and Pearson correlation coefficients, 0.68 and 0.64, respectively. NDVI demonstrated a significant association (*p* < 0.01) with 0.62 and 0.60. SRSI had a significant association with land salinity (*p* < 0.01), with 0.64 and 0.61 (Table 4). LSMI, NDVI, and SRSI were utilized separately to build land salinity monitoring models.

**Figure 2:**
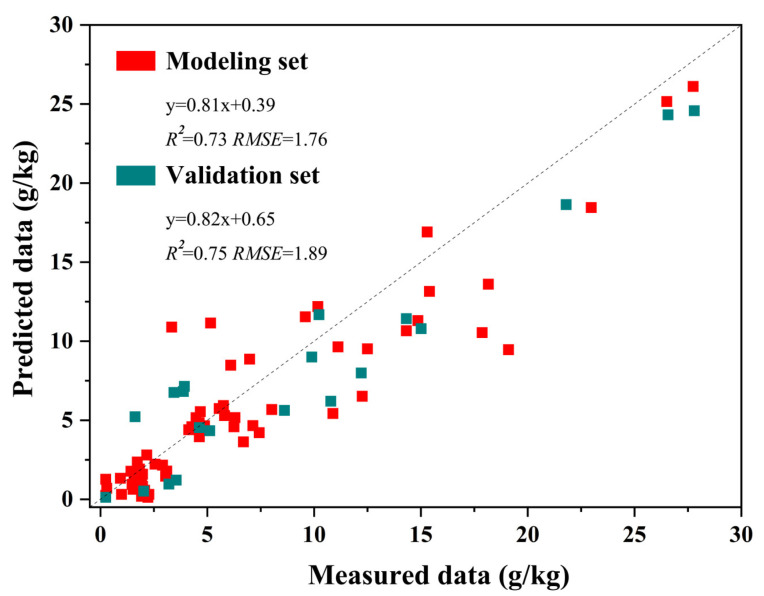
Scatter plot of the optimal model of land salinity based on the UAV image.

The random forest (RF) algorithm was then employed in this study to create retrieval models of land salinity based on the LSMI, NDVI, and SRSI [28,37,38]. The results indicated that the *R*^2^ values of the LSMI-based RF model showed stronger fitting impacts than the estimation model based on NDVI and SRSI (Table 5), which was the highest modeling and validation accuracies (*R*^2^ = 0.73 and 0.75) among the three sensitive parameters in order of modeling and validation accuracies, and the *RPD* is higher than 2 (Table 5 and Figure 2). The combination of LSMI and RF has adequate land salinity estimation capacity compared to the commonly used methods.

### 4.3. Image Correction

**Figure 3:**
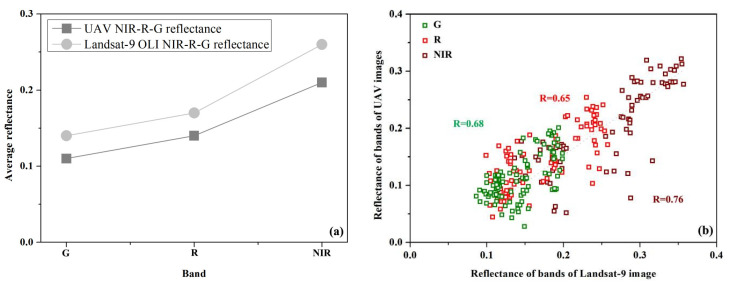
Comparison of UAV and Landsat-9 OLI images: (**a**) reflectance comparison of the G, R, NIR band; (**b**) scatter plot of G, R, and NIR reflectance of Landsat-9 image (X-axis) and UAV image (Y-axis).

The reflectance of the three sensitive bands (G, R, NIR) was compared with the reflectance of the Landsat-9 image corresponding to the study sample sites, and the results are shown in Figure 3a. The average reflectance of the three bands of the Landsat-9 image is higher than that of the corresponding UAV image band, the change trend is congruent, and the two images can be converted by the reflectance correction coefficient.

Furthermore, the reflectance of the three sensitive bands at each sampling site are plotted and shown in Figure 3b. The correlation between Landsat-9 image and NIR band reflectance is 0.76, while the G and R reflectance of Landsat-9 images exhibited moderate correlations with the corresponding UAV multi-spectral bands (0.68 and 0.65, respectively). The reflectivity correction coefficient is the ratio of the three sensitive band pixels of each sampling point of the Landsat-9 image to the average of the corresponding points of the UAV multi-spectral image (Table 6). The three sensitive reflectance correction coefficients were divided by the corresponding Landsat-9 image band to correct the Landsat-9 image, in order to achieve the subsequent land salinity monitoring in the study area.

### 4.4. Spatial Distribution of Land Salinity

**Figure 4:**
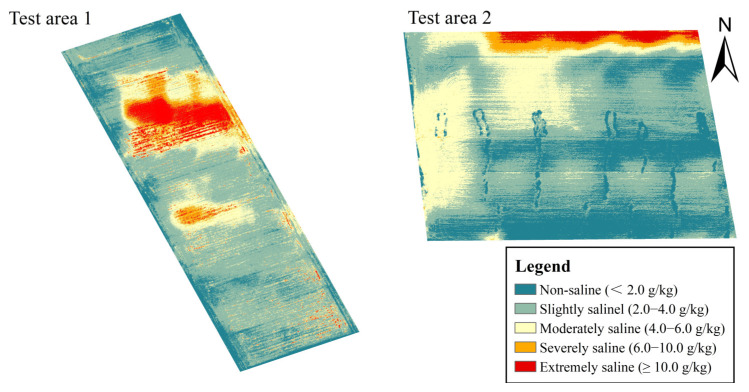
Distribution of land salinity using the proposed LSMI-based RF method in the test areas.

Land salinity in the test areas based on the proposed model was computed and shown in Figure 4. The retrieval values of soil salinity ranged from 0.43 to 20.28 g/kg, with an average value of 7.37 g/kg, which was close to the descriptive statistical results of the soil samples (Table 4). The test areas can be divided into five classes based on the saline land grading standard [39], namely extremely saline soil (salt content greater than 10.0 g/kg), severely saline soil (salt content 6.0–10.0 g/kg), moderately saline soil (salt content 4.0–6.0 g/kg), slightly saline soil (salt content 2.0–4.0 g/kg), and non-saline soil (Figure 3). According to the area calculation result, the extremely saline soil occupied the lowest share of 6.3 percent of the five grades. Severely and moderately saline soil zones accounted for 10.5 and 15.6 percent of the overall test area, respectively. The proportion of slightly saline soil was 55.4 percent, the highest of the five categories. The non-saline region encompassed 12.2 percent of the test area. The geographical analysis demonstrated that land salinization is widespread in the test areas.

**Figure 5:**
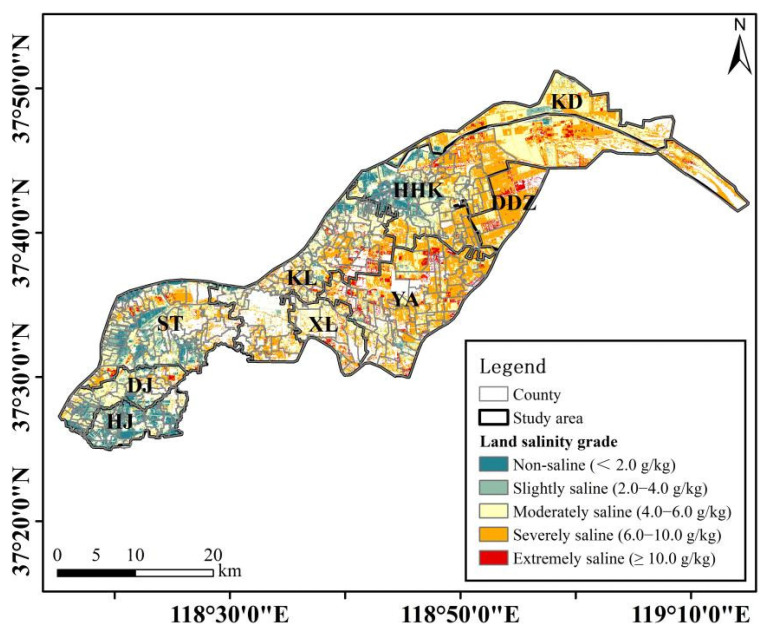
Spatial distribution of land salinity monitoring results in the cultivated land of the study area.

The results of land salinity monitoring for the study area based on the proposed model and corrected Landsat-9 image presented in Figure 5 and Table 7 provide a summary of the coverage area for each grade of land salinity. Land salinization affected 93.12% of the cultivated land in the study area, and the non-saline grade occupied only 6.88% of the total cultivated land in the study area, which was mainly located in ST (18.09 km^2^), HJ (15.84 km^2^), and HHK (15.76 km^2^). The slightly saline soil class covered an area of 99.43 km^2^ in the study area. ST (26.01 km^2^), HHK (24.91 km^2^), and HJ (16.47 km^2^) topped three among the nine towns. The moderately saline grade covered 37.76% of the total cultivated land in the study area, which was mainly located in HHK (97.48 km^2^), KL (50.13 km^2^), and ST (48.55 km^2^). The severely saline soil class was found to be the most extensive, covering 38.41% of the total cultivated land area and widely distributed throughout the study area, while HHK and YA covered 108.16 and 80.99 km^2^. The extremely saline grade (salinity content ≥10 g/kg) covered an area of 52.07 km^2^, accounting for 5.82% of the total cultivated area, which was the least among the five grades. YA and HHK contributed 16.76 and 11.41 km^2^, respectively. Overall, the salinization degree of most of the cultivated land in the study area was at moderately saline or below levels (55.76%, Table 7), while the severely saline soil grade was widely distributed throughout the study area (Figure 5).

### 4.5. Spatial Analysis of Land Salinity

**Figure 6:**
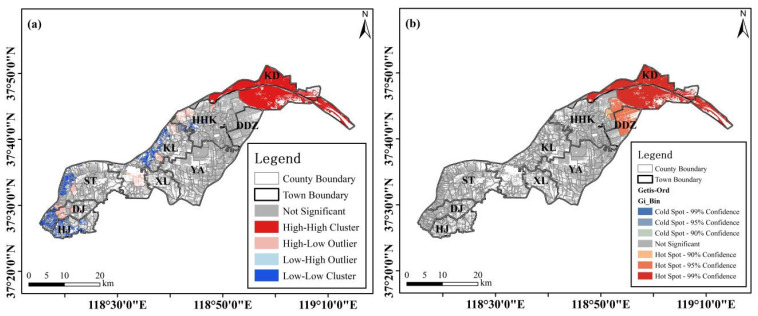
Local Moran’s I (**a**) and Getis-Ord GI* (**b**) of saline land in the study area.

Moran’s I and Getis-Ord GI* analysis was applied to the land-salinity-affected areas in 461 counties of the 9 towns (Figure 6a), as they were not applicable at the township scale. Global Moran’s I computation results demonstrated that the distribution of saline land has positive spatial autocorrelation (0.311, *p* = 0.000). Local Moran’s I analysis showed that non-significant cluster types prevailed in the study area (332 of 461 counties), which were distributed in all the 9 study towns. Conversely, there were only five counties exhibiting a high–high cluster type, which were located in KD (2), HHK (2), and DDZ (1). A total of 19 counties in 8 towns (except DDZ) showed a high–low cluster type. In this category, DJ, HHK, and KL contain four counties, and ST has three counties. Only one county in KD displayed a low–high cluster type. Different from the other three types, 104 counties showed a low–low type in 5 towns (DJ, HJ, HHK, KL, and ST), among which DJ (26) and HJ (22), and KL (22), ST (22) ranked first and second, respectively (Table 8).

The Getis-Ord Gi* analysis was used to detect cold and hot spots of apple orchard land areas in the study area. Figure 6b shows whether the spatial clustering of the land salinity was significant and, if so, at what level (0.01, 0.05, and 0.1 levels). The spatial weight matrix was computed based on the Euclidean distance between sampling sites, and the distance threshold was 5829.72 m. The spatial heterogeneity analysis found that there were two 0.1-significant-level hot spots located in HHK, eight 0.05-significant-level hot spots located in HHK (4) and DDZ (4), and ten 0.01-significant-level hot spots located in HHK (4), KD (4), and DDZ (2). The distribution patterns of hot spots converged with those of the local Moran index computation results.

**Figure 7:**
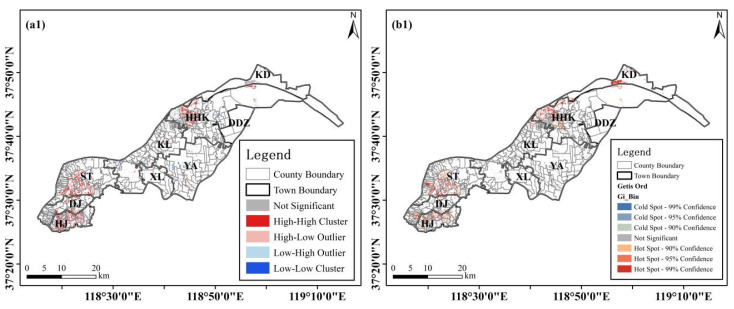

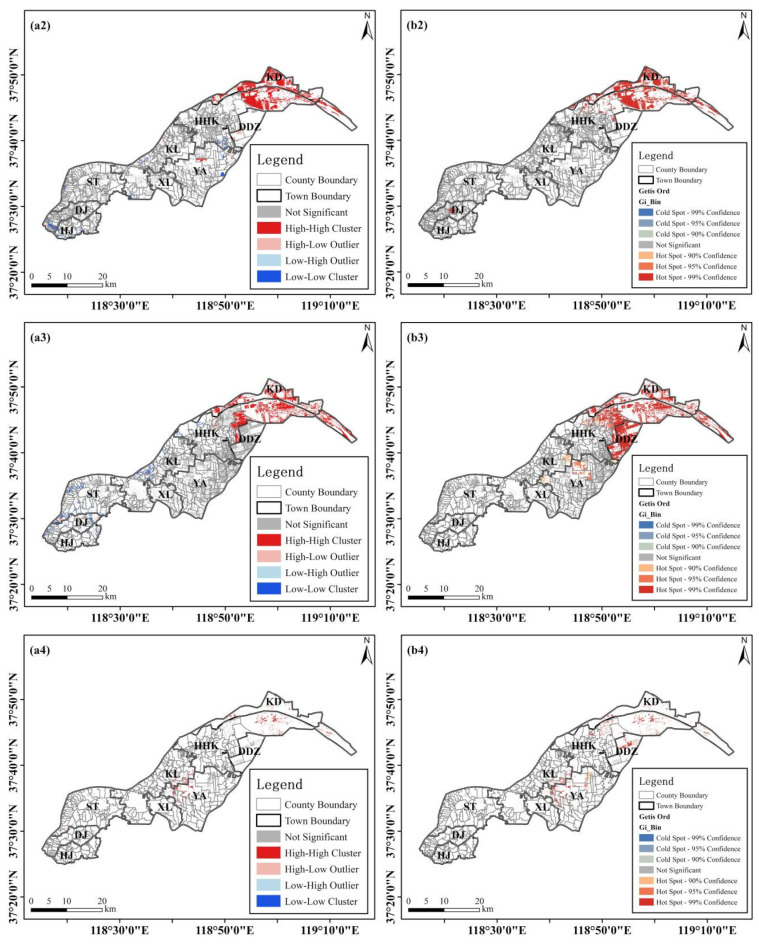
Local Moran’s I (**a1**–**a4**) and Getis-Ord GI* (**b1**–**b4**) of different land salinity levels. (**a1**,**b1**): slightly saline grade, (**a2**,**b2**) moderately saline grade, (**a3**,**b3**) severely saline grade, and (**a4**,**b4**) extremely saline grade.

To further understand the spatial characteristics of the different levels of land salinization in the study area, the results of local Moran’s I and Getis-Ord Gi* analysis of slightly saline grade (salt content 2.0–4.0 g/kg, Figure 7(a1,b1)), moderately saline grade (salt content 4.0–6.0 g/kg, Figure 7(a2,b2)), severely saline grade (salt content greater than 10.0 g/kg, Figure 7(a3,b3)), and extremely saline grade (salt content greater than 10.0 g/kg, Figure 7(a4,b4)) were separately computed and depicted. The summarized results are shown in Table 9.

The spatial characteristics of different salinity grades varied significantly. Specifically, the slightly saline grade exhibited a distinct high–high cluster type (Figure 7(a1) and Table 9). Among the 177 cluster-type counties, there were 47 high–high cluster counties distributed in HJ (16), HHK (12), ST (12), and DJ (7), accounting for 26.55% of the total. On the other hand, the high–low, low–high, and low–low types were predominantly found in KL, ST, and KL town respectively. Regarding the moderately, severely, and extremely saline grades, they all displayed a significant high–high cluster type primarily in HHK and KL town, as depicted in Figure 7(a2, a3, a4). Notably, KD town was unique as it contained a low–high cluster type for moderately to extremely saline grades (Table 9). In conclusion, conducting separate spatial analyses is recommended for subsequent studies due to variations observed across different salinity levels.

## 5. Discussion

This study proposed an index-based method to accurately estimate land salinity content using UAV and the Landsat-9 multi-spectral image framework. Results found that the proposed method can accurately estimate land salinity content with the modeling *R*^2^ and *RMSE* of 0.73 and 1.76 and the validation *R*^2^, *RMSE*, and *RPD* of 0.75, 1.89, and 2.11, respectively. The salinization degree of most of the cultivated land was at the moderate or below levels (55.76%), while the severely saline soil grade (with a salinity content of 6–8 g/kg) covered 38.41% of the total cultivated land area and was widely distributed throughout the study area. The distribution of saline land has positive spatial autocorrelation (0.311, *p* = 0.000). High–high cluster types occurred mainly in the Kendong and Huanghekou towns (80%), and the low–low cluster type was found mainly in the Dongji, Haojia, Kenli, and Shengtuo towns (88.46%). The spatial characteristics of different salinity grades varied significantly, so conducting separate spatial analyses is recommended for subsequent studies.

According to the results of the spectral screening analysis, significant correlation links were observed between soil salinity and visible (G, R) as well as NIR bands. The study found that the primary minerals responsible for land salinization in the study area are rock salt and gypsum, with Cl^−^ and SO_4_^2−^ being the main anions and Na^+^ and Ca^2+^ being the main cations [40]. Another research demonstrated that gypsum exhibits molecular vibration absorption spectrum characteristics in the NIR range, and that both visible and NIR bands can be utilized to collect spectral information on SO_4_^2−^ [41]. Additionally, studies have indicated that saline soil displays higher reflectance in the visible and NIR ranges compared to non-saline land [42]. Therefore, the proposed index is reliable for predicting land salinity content.

Compared to existing studies, this study found a weak correlation between the reflectance of the red-edge band and land salinity content. Since its launch in 2015, Sentinel-2 imagery has been utilized for regional land salinization analysis due to its relatively higher spatial resolution (10 m) compared to Landsat-8/9 (15 m after fusion). Furthermore, with three red-edge bands available, Sentinel-2 imagery can better utilize vegetation information for retrieving land salinization content. In this study, it is found that a high-precision land salinization monitoring model can be constructed without considering the red-edge band. Considering the wider temporal coverage of Landsat images (from 1972 to the present), the Landsat series image has the potential to be used as the main data source for land salinization monitoring. Further studies can use Landsat images and the proposed method in this study to monitor the evolution of land salinization in the study area in the recent 50 years.

Based on the spatial analysis results of land salinization obtained in this study, low degrees of land salinity were found in HJ, KL, and ST in the southwest of the study area, and saline land areas were distributed in the study area and prevailed in coastal towns, e.g., HHK and YA. HJ, KL, and ST are relatively far from the sea, and the freshwater resources of the Yellow River, crop planting, and drainage practices jointly mitigate land salinization [43]. This also explains why HHK and KD also contained low-salinity areas. Conversely, the northeast coastal area (KD and HHK) is plagued by severe and extreme salinization, which is in line with previous research findings [44]. These regions were primarily influenced by factors such as low elevation, intrusion of seawater, and facile accumulation of salt on the soil surface. Due to inadequate conditions for agricultural development, it is recommended to plan rationally for fishery and aquaculture activities [45].

The spatial distribution analysis found that 93.12% of the cultivated land in the study area was affected by land salinization, and the severely saline soil grade covered 38.41% of the total cultivated land area and was widely distributed throughout the study area. Therefore, targeted improvement and treatment measures should be implemented to combat land salinity. In areas affected by seawater intrusion, it is imperative to reinforce drainage systems to prevent or mitigate the upward migration of salinity [46]. In areas with high land salinity, proper soil management is crucial. Field organization and timely deep loosening and smoothing of the soil are recommended. Additionally, covering the surface of cultivated land with straw can reduce evaporation by creating a residual layer that improves land salinization [47,48]. In the course of agricultural production, it is imperative to conserve water resources and adopt rational irrigation practices. To mitigate cultivated land salinization, micro-irrigation systems, agricultural channel laying, and concealed pipe alkali drainage should be considered [49]. For areas in the east of the study area, the extremely saline land can be planed for fishery and aquaculture activities [45].

This study proposed a scale-up method to retrieve land salinity in China’s typical coastal area. However, it has limitations. Due to the limited spectral penetration ability, soil samples were only collected from the surface layer (0–10 cm). For the purpose of agriculture and food security, more attention should be given to indirect approaches for assessing root-zone salinization (0–100 cm) [50]. Moreover, this study estimated land salinity in the Kenli district. Considering the current severe land salinity situation in the Yellow River Delta, future research will focus on the estimation and modeling of land salinity in the entire Yellow River Delta to provide theoretical and methodological support for the formulation and implementation of regional governance policies.

## 6. Conclusions

This study proposed a land salinity monitoring index to accurately retrieve land salinity using the harmonized UAV and Landsat-9 multi-spectral dataset. Results found the proposed method can accurately estimate the land salinity content in the study area. The salinization degree of most of the cultivated land was at moderate or below levels, while the severely saline land was widely distributed throughout the study area. The distribution of saline land showed positive spatial autocorrelation. The spatial characteristics of different salinity grades varied significantly, so conducting separate spatial analyses is recommended for subsequent studies. Future research will be conducted to investigate land salinity across the Yellow River Delta to provide theoretical and methodological support for the development and implementation of regional governance policies.

## Figures and Tables

**Table 1:** Band information of UAV and Landsat-9 OLI multi-spectral sensors (550–865 nm).

ID	Band	Abbreviation	Center Wavelength (nm) of Sequoria UAV Image	Center Wavelength (nm) of Landsat-9 OLI Image
1	Green	G	550	563
2	Red	R	660	655
3	Red-edge	REG	735	-
4	Near-infrared	NIR	790	865

**Table 2:** Algebraic computations of UAV multi-spectral data.

ID	Transformation	Equation
1	Addition	R + G, G + NIR, NIR + R
2	Subtraction	R−G, G−NIR, NIR−R
3	Division	R/G, R/NIR, G/R, G/NIR, NIR/R, NIR/G
4	Logarithmic	Lg(R), Lg(G), Lg(B)
5	Reciprocal	1/R, 1/G, 1/NIR
6	Ratio	|(R−G)/(R + G)|, |(R−NIR)/(R + G)|, |(G−NIR)/(R + G)|; |(R−G)/(G + NIR)|, |(R−NIR)/(G + NIR)|,|(G−NIR)/(G + NIR)|; |(R−G)/(NIR + R)|, |(R−NIR)/(NIR + R)|, |(G−NIR)/(NIR + R)|

G, R, and NIR are the reflectance of the green, red, and near-infrared band of the UAV image, respectively.

**Table 3:** Sensitive parameters (|R| > 0.45) of the G, R, NIR.

ID	Land Salinity Monitoring Models	|R|
1	G	0.49
2	R	0.45
3	NIR	0.51
4	G/R	0.48
5	NIR/R	0.58
6	NIR/G	0.52
7	|(R − NIR)/(NIR + R)|	0.60
8	|(R − G)/(R + G)|	0.51
9	|(R − G)/(NIR + R)|	0.56
10	|(R − NIR)/(NIR + G)|	0.63

**Table 4:** Correlation analysis of sensitive spectral index with land salinity.

Spectral Index	Gray Correlation Coefficient	Pearson Correlation Coefficient
LSMI	0.68 **	0.64 **
NDVI	0.62 **	0.60 **
SRSI	0.64 **	0.61**

** significant at 0.01 level.

**Table 5:** Accuracy statistical results of LSMI, SRSI and NDVI-based RF models.

Modeling Method	Modeling Accuracy		Validation Accuracy		
	*R* ^2^	*RMSE*	*R* ^2^	*RMSE*	*RPD*
LSMI	0.73	1.76	0.75	1.89	2.11
SRSI	0.66	2.54	0.69	2.44	1.88
NDVI	0.65	2.97	0.63	2.79	1.45

**Table 6:** Reflectance correction coefficient of the Landsat-9 satellite image.

Band	G	R	NIR
Reflectance correction coefficient	1.32	1.25	1.05

**Table 7:** Areas of different land salinity grades in the study area.

Town	Land Salinity Grade					Total (km^2^)
	Non-Saline	Slightly Saline	Moderately Saline	Severely Saline	Extremely Saline	
DDZ	0	0.01	5.10	35.63	5.21	45.95
DJ	5.74	10.62	29.13	7.10	1.08	53.67
HJ	15.84	16.47	12.30	1.59	0.13	46.33
HHK	15.76	24.91	97.48	108.16	11.41	257.72
KD	0.84	4.54	40.37	27.28	1.74	74.77
KL	4.60	11.34	50.13	44.12	9.31	119.5
ST	18.09	26.01	48.55	26.35	4.01	123.01
XL	0.02	0.63	14.97	12.33	2.42	30.37
YA	0.66	4.90	39.71	80.99	16.76	143.02
Total (km^2^)	61.55	99.43	337.74	343.55	52.07	894.34

**Table 8:** Cluster types using local Moran’s I in the study area.

Town	Cluster Type					Total
	Not Significant	H-H	H-L	L-H	L-L	
DDZ	12	1	/	/	/	13
DJ	21	/	4	/	26	51
HJ	18	/	1	/	22	41
HHK	75	2	4	/	12	93
KD	4	2	1	1	/	8
KL	66	/	4	/	22	92
ST	47	/	3	/	22	72
XL	23	/	1	/	/	24
YA	66	/	1	/	/	67
Total	332	5	19	1	104	461

H-H: high–high cluster, H-L: high–low cluster; L-H: low–high cluster; L-L: low–low cluster.

**Table 9:** Cluster types of the four land saline grades.

a. Cluster Types of Slightly Saline Grade						b. Cluster Types of Moderately Saline Grade				
Town	Cluster type				Total	Cluster type				Total
	H-H	H-L	L-H	L-L		H-H	H-L	L-H	L-L	
DDZ	/	/	/	/	0	/	/	/	/	0
DJ	7	/	6	/	13	/	1	/	9	10
HJ	16	/	6	/	22	/		/	5	5
HHK	12	/	5	14	31	1	1	/	11	13
KD	/	/	/	/	0	1	/	1	/	2
KL	/	7	/	37	44	/	1	/	6	7
ST	12	/	10	/	22	/	/	/	3	3
XL	/	/	/	9	9	/	/	/	/	0
YA	/	2	/	34	36	1	2	/	3	6
Total	47	9	27	94	177	3	5	1	37	46
**c. Cluster types of severely saline grade**						**d. Cluster types of extremely saline grade**				
Town	Cluster type				Total	Cluster type				Total
	H-H	H-L	L-H	L-L		H-H	H-L	L-H	L-L	
DDZ	/	/	/	/	0	/	/	/	/	0
DJ	/	1	/	19	20	/	1	/	19	20
HJ	/	/	/	7	7	/	/	/	7	7
HHK	1	/	/	/	1	1	/	/	/	1
KD	1	/	1	/	2	1	/	1	/	2
KL	4	1	/	14	19	4	1	/	14	19
ST	/	1	/	23	24	/	1	/	23	24
XL	/	/	/	1	1	/	/	/	1	1
YA	4	/	1	1	6	4	/	1	1	6
Total	10	3	2	65	80	10	3	2	65	80

H: high–high cluster, H-L: high–low cluster; L-H: low–high cluster; L-L: low–low cluster.

## Data Availability

Data used in this study are available under request.

## References

[1] Carlucci M., Salvia R., Quaranta G., Salvati L., Imbrenda V. (2022). Official statistics, spatio-temporal dynamics and local-scale monitoring: Toward integrated environmental-economic accounting for land degradation. Lett. Spat. Resour. Sci..

[2] Singh A. (2022). Soil salinity: A global threat to sustainable development. Soil Use Manag..

[3] Clinton N., Stuhlmacher M., Miles A., Aragon N.U., Wagner M., Georgescu M., Herwig C., Gong P. (2018). A global geospatial ecosystem services estimate of urban agriculture. Earth’s Future.

[4] Ivushkin K., Bartholomeus H., Bregt A.K., Pulatov A., Kempen B., De Sousa L. (2019). Global mapping of soil salinity change. Remote Sens. Environ..

[5] Okur B., Örçen N. (2020). Soil salinization and climate change. Climate Change and Soil Interactions.

[6] Wu J., Li P., Qian H., Fang Y. (2014). Assessment of soil salinization based on a low-cost method and its influencing factors in a semi-arid agricultural area, northwest China. Environ. Earth Sci..

[7] Yu J., Li Y., Han G., Zhou D., Fu Y., Guan B., Wang G., Ning K., Wu H., Wang J. (2014). The spatial distribution characteristics of soil salinity in coastal zone of the Yellow River Delta. Environ. Earth Sci..

[8] Su Y., Li T., Cheng S., Wang X. (2020). Spatial distribution exploration and driving factor identification for soil salinisation based on geodetector models in coastal area. Ecol. Eng..

[9] Fang S., Tu W., Mu L., Sun Z., Hu Q., Yang Y. (2019). Saline alkali water desalination project in Southern Xinjiang of China: A review of desalination planning, desalination schemes and economic analysis. Renew. Sustain. Energy Rev..

[10] Xue J., Huo Z., Wang F., Kang S., Huang G. (2018). Untangling the effects of shallow groundwater and deficit irrigation on irrigation water productivity in arid region: New conceptual model. Sci. Total Environ..

[11] Han D., Song X., Currell M.J., Cao G., Zhang Y., Kang Y. (2011). A survey of groundwater levels and hydrogeochemistry in irrigated fields in the Karamay Agricultural Development Area, northwest China: Implications for soil and groundwater salinity resulting from surface water transfer for irrigation. J. Hydrol..

[12] Minhas P.S., Ramos T.B., Ben-Gal A., Pereira L.S. (2020). Coping with salinity in irrigated agriculture: Crop evapotranspiration and water management issues. Agric. Water Manag..

[13] Hailu B., Mehari H. (2021). Impacts of soil salinity/sodicity on soil-water relations and plant growth in dry land areas: A review. J. Natural Sci. Res..

[14] Mau Y., Porporato A. (2015). A dynamical system approach to soil salinity and sodicity. Adv. Water Resour..

[15] Keesstra S., Mol G., De Leeuw J., Okx J., Molenaar C., De Cleen M., Visser S. (2018). Soil-related sustainable development goals: Four concepts to make land degradation neutrality and restoration work. Land.

[16] Sharma P.K., Kumar D., Srivastava H.S., Patel P. (2018). Assessment of different methods for soil moisture estimation: A review. J. Remote Sens. GIS.

[17] Gorji T., Yildirim A., Hamzehpour N., Tanik A., Sertel E. (2020). Soil salinity analysis of Urmia Lake Basin using Landsat-8 OLI and Sentinel-2A based spectral indices and electrical conductivity measurements. Ecol. Indic..

[18] Allbed A., Kumar L. (2013). Soil salinity mapping and monitoring in arid and semi-arid regions using remote sensing technology: A review. Adv. Remote Sens..

[19] Azabdaftari A., Sunar F. Soil salinity mapping using multitemporal Landsat data. Proceedings of the International Archives of the Photogrammetry, Remote Sensing and Spatial Information Sciences.

[20] Morgan R.S., El-Hady M.A., Rahim I.S. (2018). Soil salinity mapping utilizing sentinel-2 and neural networks. Indian J. Agric. Res..

[21] Wang J., Peng J., Li H., Yin C., Liu W., Wang T., Zhang H. (2021). Soil salinity mapping using machine learning algorithms with the Sentinel-2 MSI in arid areas, China. Remote Sens..

[22] Ge X., Ding J., Teng D., Wang J., Huo T., Jin X., Wang J., He B., Han L. (2022). Updated soil salinity with fine spatial resolution and high accuracy: The synergy of Sentinel-2 MSI, environmental covariates and hybrid machine learning approaches. Catena.

[23] Kaplan G., Gašparović M., Alqasemi A.S., Aldhaheri A., Abuelgasim A., Ibrahim M. (2023). Soil salinity prediction using Machine Learning and Sentinel–2 Remote Sensing Data in Hyper–Arid areas. Phys. Chem. Earth Parts A/B/C.

[24] Alamdar S., Ghazban F., Zarei A. (2023). Efficiency of Machine Learning Algorithms in Soil Salinity Detection Using Landsat-8 OLI Imagery. ISPRS Ann. Photogramm. Remote Sens. Spat. Inf. Sci..

[25] Ivushkin K., Bartholomeus H., Bregt A.K., Pulatov A., Franceschini M.H.D., Kramer H., van Loo E.N., Roman V.J., Finkers R. (2019). UAV based soil salinity assessment of cropland. Geoderma.

[26] Zhao W., Zhou C., Zhou C., Ma H., Wang Z. (2022). Soil salinity inversion model of oasis in arid area based on UAV multispectral remote sensing. Remote Sens..

[27] Yang N., Yang S., Cui W., Zhang Z., Zhang J., Chen J., Ma Y., Lao C., Song Z., Chen Y. (2021). Effect of spring irrigation on soil salinity monitoring with UAV-borne multispectral sensor. Int. J. Remote Sens..

[28] Yu X., Chang C., Song J., Zhuge Y., Wang A. (2022). Precise monitoring of soil salinity in China’s Yellow River Delta using UAV-borne multispectral imagery and a soil salinity retrieval index. Sensors.

[29] Xie L., Feng X., Zhang C., Dong Y., Huang J., Cheng J. (2022). A Framework for Soil Salinity Monitoring in Coastal Wetland Reclamation Areas Based on Combined Unmanned Aerial Vehicle (UAV) Data and Satellite Data. Drones.

[30] Qi G., Chang C., Yang W., Gao P., Zhao G. (2021). Soil salinity inversion in coastal corn planting areas by the satellite-UAV-ground integration approach. Remote Sens..

[31] Matarira D., Mutanga O., Naidu M. (2022). Performance evaluation of pansharpening Sentinel 2A imagery for informal settlement identification by spectral-textural features. Trans. R. Soc. S. Afr..

[32] Terhoeven-Urselmans T., Schmidt H., Joergensen R.G., Ludwig B. (2008). Usefulness of near-infrared spectroscopy to determine biological and chemical soil properties: Importance of sample pre-treatment. Soil Biol. Biochem..

[33] Chen H., Ma Y., Zhu A., Wang Z., Zhao G., Wei Y. (2021). Soil salinity inversion based on differentiated fusion of satellite image and ground spectra. Int. J. Appl. Earth Obs. Geoinf..

[34] Moran P.A. (1950). Notes on continuous stochastic phenomena. Biometrika.

[35] Anselin L. (1995). Local indicators of spatial association—LISA. Geogr. Anal..

[36] Getis A., Ord J.K. (2010). The analysis of spatial association by use of distance statistics. Perspectives on Spatial Data Analysis.

[37] Flores J.L.G., Rodríguez M.R., Jiménez A.G., Farzamian M., Galán J.F.H., Bellido B.S., Sacristan P.C., Vanderlinden K. (2022). Depth-Specific Soil Electrical Conductivity and NDVI Elucidate Salinity Effects on Crop Development in Reclaimed Marsh Soils. Remote Sens..

[38] Yu H., Liu M., Du B., Wang Z., Hu L., Zhang B. (2018). Mapping soil salinity/sodicity by using Landsat OLI imagery and PLSR algorithm over semiarid West Jilin Province, China. Sensors.

[39] Hu K., Wang S., Li H., Huang F., Li B. (2014). Spatial scaling effects on variability of soil organic matter and total nitrogen in suburban Beijing. Geoderma.

[40] An L.S., Zhao Q.S., Ye S.Y. (2011). Water-salt interactions factors and vegetation effects in the groundwater ecosystem in Yellow River Delta. Adv. Water Sci..

[41] Xu W. (2018). Spectral Discriminant Analysis of Martian Simulated Minerals and Brines.

[42] Fan X., Liu Y., Tao J., Weng Y. (2015). Soil salinity retrieval from advanced multi-spectral sensor with partial least square regression. Remote Sens..

[43] Wang Z., Zhao G., Gao M. (2015). Spatial differentiation of soil water and salt in spring in typical Yellow River Delta region—A case study of Kenli County. Agric. Environ. Dev..

[44] Wang N., Qi W., Wang D. (2012). Spatial variation of soil nutrients and salinity in coastal saline-alkali soil based on transect. Chin. J. Appl. Ecol..

[45] Tian P., Liu Y., Li J., Pu R., Cao L., Zhang H., Ai S., Yang Y. (2022). Mapping Coastal Aquaculture Ponds of China Using Sentinel SAR Images in 2020 and Google Earth Engine. Remote Sens..

[46] Feng Y., Li S., Lu Y. (2013). Sequence stratigraphy and architectural variability in late Eocene lacustrine strata of the Dongying Depression, Bohai Bay Basin, eastern China. Sediment. Geol..

[47] Zhu W., Yang J., Yao R., Wang X., Xie W., Shi Z. (2021). Buried layers change soil water flow and solute transport from the Yellow River Delta, China. J. Soils Sediments.

[48] Long X.H., Liu L.P., Shao T.Y., Shao H.B., Liu Z.P. (2016). Developing and sustainably utilize the coastal mudflat areas in China. Sci. Total Environ..

[49] Singh A. (2021). Soil salinization management for sustainable development: A review. J. Environ. Manag..

[50] Scudiero E., Skaggs T., Anderson R., Corwin D. Soil degradation in farmlands of California’s San Joaquin Valley resulting from drought-induced land-use changes. Proceedings of the EGU General Assembly Conference.

